# Identification of a Pathogenic Mutation of the Lipase Maturation Factor 1 (LMF1) Gene Causing Recurrent Pancreatitis and Requiring Critical Care

**DOI:** 10.3390/jcm14061827

**Published:** 2025-03-08

**Authors:** Andrés Fernando Montalvo, Fabricio González-Andrade, María José Molestina, Jhonny Manuel Carranza, Claudio Maldonado, Denise Battaglini, Jorge Luis Vélez-Paéz

**Affiliations:** 1 Hospital Provincial Pablo Arturo Suarez, Ángel Ludeña y Machala Oe5261, Quito 170301, Ecuador; amontalvo4@hotmail.com (A.F.M.); majomolestina83@gmail.com (M.J.M.); jhonnycarranza87@gmail.com (J.M.C.); jorgeluisvelez13@hotmail.com (J.L.V.-P.); 2 Facultad de Ciencias de la Salud, Dirección de Posgrados, Universidad Tecnológica Indoamérica, Calle Machala y Sabanilla, Quito 170301, Ecuador; 3 SYNLAB-Ecuador, Calle Oe7A N31-145 y Av. Mariana de Jesús, Quito 170129, Ecuador; claudio.maldonado@synlab.ec; 4 Department of Surgical Sciences and Integrated Diagnostics, University of Genova, 16126 Genova, Italy; battaglini.denise@gmail.com; 5 Anesthesia and Intensive Care, IRCCS Ospedale Policlinico San Martino, 16132 Genova, Italy; 6 Facultad de Ciencias Médicas, Unidad de Medicina Traslacional, Universidad Central del Ecuador, Iquique N14-121 y Sodiro-Itchimbía, Quito 170403, Ecuador

**Keywords:** rare disease, familial chylomicronemia syndrome, hyperlipoproteinemia, combined lipase deficiency, lipoprotein lipase deficiency, pancreatitis

## Abstract

**Background:** Familial chylomicronemia syndrome (FCS) is a rare autosomal recessive disorder characterized by extreme hypertriglyceridemia (>1000 mg/dL), recurrent pancreatitis, and lipoprotein lipase (LPL) deficiency. Mutations in the LMF1 gene, encoding a chaperone protein required for LPL maturation, can lead to combined lipase deficiency. This study reports a case of a 33-year-old Ecuadorian mestizo woman presenting with recurrent pancreatitis secondary to severe hypertriglyceridemia, in whom two LMF1 variants of uncertain significance (VUS) were identified. **Methods:** Whole-exome sequencing (WES) was performed on the patient and her asymptomatic son using next-generation sequencing (NGS). Data analysis included computational pathogenicity predictors (REVEL, PolyPhen, SIFT, MutationTaster, etc.). Two LMF1 variants—c.1142C>T (p.Pro381Leu) and c.897G>A (p.Gln299Gln)—were identified. Their pathogenic potential was assessed based on allele frequency (gnomAD), bioinformatics predictions, and ACMG criteria. **Results:** Both variants were classified as VUS, with c.897G>A predicted to affect splicing, potentially leading to loss of function. The c.1142C>T (p.Pro381Leu) variant, despite its low frequency, remains unclassified due to insufficient evidence. The patient’s son carried one variant but was asymptomatic. The patient’s phenotype suggested an intermediate form between monogenic and polygenic hypertriglyceridemia. **Conclusions:** This is a new Ecuadorian report of LMF1-related hypertriglyceridemia, highlighting the need for functional studies to confirm pathogenicity. Given the classification of both variants as VUS, further research is required to elucidate their clinical significance. This case underscores the necessity of a combined genetic and biochemical approach for diagnosing and managing severe hypertriglyceridemia.

## 1. Introduction

Chylomicronemia describes a severe form of exogenous hypertriglyceridemia (HTG), characterized by triglyceride (TG) levels exceeding 1000 mg/dL (11.3 mmol/L). This condition is associated with a significantly increased risk of pancreatitis due to the accumulation of chylomicrons in the bloodstream [1]. While chylomicronemia can, in rare cases, result from monogenic disorders that drastically reduce the enzymatic activity of lipoprotein lipase (LPL), most cases arise from polygenic factors combined with additional triggers. The monogenic form of the disease, known as familial chylomicronemia syndrome (FCS), is rare and primarily caused by mutations in LPL, impairing the clearance of triglyceride-rich lipoproteins (TRLs) [2]. In contrast, multifactorial chylomicronemia syndrome (MCS) is far more common and occurs in individuals with a genetic predisposition to polygenic HTG, with TG levels typically ranging between 500 and 999 mg/dL. These patients may develop severe chylomicronemia in response to factors such as comorbidities or the use of certain medications [3]. In both cases, the greatest clinical risk is acute pancreatitis, which can be prevented by maintaining TG levels below 500 mg/dL.

LPL plays a central role in triglyceride metabolism. It is primarily synthesized in adipocytes and myocytes, where it undergoes a maturation process mediated by lipase maturation factor 1 (LMF1) before being secreted into the interstitial space. From there, it is transported across the endothelium, where it binds to the glycosylphosphatidylinositol-anchored high-density lipoprotein-binding protein 1 (GPIHBP1) in the endothelial lumen. This interaction allows LPL to act on circulating TRL, facilitating triglyceride hydrolysis and clearance from plasma [4]. LPL activity is regulated by several triglyceride-rich apolipoproteins, such as APOC2, an essential cofactor for its activation; APOA5, which stabilizes the LPL-TRL complex and enhances triglyceride hydrolysis; and APOC3, which inhibits LPL activity [5].

Since TG molecules are transported in plasma through different lipoproteins, it is important to differentiate between hypertriglyceridemia caused by intestinal chylomicrons and that resulting from hepatically secreted very low-density lipoproteins (VLDLs). Chylomicrons transport apolipoprotein B-48 (apoB-48) and are involved in dietary fat transport, whereas VLDL contains apolipoprotein B-100 (apoB-100) and originates in the liver. Measuring apoB levels can help distinguish between these two types of dyslipidemia, as apoB-100 is characteristic of VLDL, while apoB-48 is a key marker of chylomicrons in FCS [6].

Chylomicronemia syndrome can have either a monogenic or polygenic genetic basis. In the case of FCS, it is an autosomal recessive disorder caused by biallelic mutations in key genes involved in triglyceride metabolism. The most affected gene is LPL, accounting for approximately 60–80% of FCS cases, although mutations have also been identified in other genes, such as APOC2, APOA5, GPIHBP1, and LMF1 [7]. In contrast, MCS is far more prevalent, affecting approximately 1 in 600 individuals. This syndrome results from a combination of multiple genetic variants affecting LPL function and its regulation, including rare heterozygous mutations in the LPL, APOC2, APOA5, GCKR, LMF1, and GPIHBP1 genes [7]. Unlike FCS, MCS is exacerbated by secondary factors such as uncontrolled diabetes mellitus, obesity, chronic kidney disease, excessive alcohol consumption, pregnancy, and the use of certain medications, including diuretics, non-selective beta-blockers, corticosteroids, immunosuppressants, antipsychotics, antidepressants, retinoids, L-asparaginase, and propofol [8,9].

Among monogenic causes of chylomicronemia, mutations in LMF1 are particularly rare. This gene encodes a transmembrane protein that acts as a critical chaperone for LPL maturation and dimerization in the endoplasmic reticulum [10]. Mutations in LMF1 are typically found in a homozygous or compound heterozygous state and lead to a deficiency in LPL activity, resulting in severe hypertriglyceridemia. Some cases of severe hypertriglyceridemia have been suggested to have a digenic origin, involving mutations in both LMF1 and LPL [9]. Due to the low prevalence of such cases, clinical reports of severe hypertriglyceridemia caused by LMF1 mutations remain extremely limited [10,11].

Chylomicronemia is a severe metabolic disorder that significantly increases the risk of acute pancreatitis. While FCS is a rare monogenic disease, MCS is a more common polygenic condition that is often exacerbated by metabolic and pharmacological factors. Mutations in LMF1 represent an exceptionally rare cause of severe hypertriglyceridemia, and identifying these cases through genetic studies is crucial for appropriate clinical management. Further research is needed to better understand the clinical spectrum of chylomicronemia associated with LMF1 and to develop optimal therapeutic strategies for these patients.

In this context, we present the case of a 33-year-old Ecuadorian mestizo woman who developed severe acute pancreatitis associated with extreme hypertriglyceridemia. Genetic studies identified a heterozygous mutation in the LMF1 gene, suggesting a rare form of hypertriglyceridemia related to LMF1. This case highlights the importance of genetic analysis in patients with severe hypertriglyceridemia and suggests that multiple genetic factors may contribute to chylomicronemia development. Additionally, it underscores the need for targeted therapeutic strategies to manage these patients and reduce the risk of severe complications such as pancreatitis.

## 2. Methods

The patient was diagnosed and managed in the Intensive Care Unit (ICU) of Pablo Arturo Suárez Hospital in Quito, Ecuador, in 2022. On admission, the patient required endotracheal intubation, invasive mechanical ventilation, and vasopressor support due to hemodynamic instability. The diagnosis of acute pancreatitis was established based on the American Gastroenterological Association (AGA) criteria, which require the presence of at least two of the following three parameters: (1) characteristic epigastric pain, (2) serum amylase and/or lipase levels elevated to more than three times the upper limit of normal, and (3) radiological findings consistent with pancreatitis on cross-sectional imaging. The patient presented with severe abdominal pain, an amylase level of 480 mg/dL, and a lipase level of 984 mg/dL. Contrast-enhanced computed tomography (CT) of the abdomen, performed within 72 h of admission, revealed pancreatic enlargement with heterogeneous attenuation, marked peripancreatic fat stranding, and a hypodense lesion with irregular margins measuring 16 × 9 mm in the pancreatic tail, comprising approximately 5% necrotic area, along with peripancreatic fluid collections. The findings were consistent with Balthazar grade D pancreatitis. Given the clinical presentation and severe hypertriglyceridemia, a diagnostic workup for familial chylomicronemia syndrome (FCS) was performed in accordance with established diagnostic algorithms [12]. The diagnosis was further supported by the Diagnostic Index of Suspicion for FCS (see Table 1).

Whole-exome sequencing (WES) was performed using next-generation sequencing (NGS) technology on the Illumina platform, with target enrichment achieved using the SureSelect QXT Target Enrichment Kit (CCP17 library). A total of 15,466 genomic variants were identified in comparison to the reference human genome. Variant prioritization was conducted using a pathogenicity-based filtering strategy, with emphasis on loss-of-function (LoF) variants (including nonsense, frameshift, and canonical splicing-site mutations), as well as variants associated with the patient’s clinical phenotype according to the Human Phenotype Ontology (HPO). The primary HPO terms applied were HP:0001733 (pancreatitis) and HP:0003119 (abnormal circulating lipid concentration). After filtering, 181 candidate variants were identified, of which two were classified as variants of uncertain significance (VUS). These variants were further analyzed in correlation with the patient’s clinical phenotype and genetic data from her son. The mean sequencing coverage was 112.7×, with 97.22% of target regions achieving ≥20× coverage, ensuring high-quality variant detection.

The genetic analysis focused on two main categories. First, genes implicated in lipid metabolism and relevant to the patient’s medical history, including ABCA1, ABCG5, ABCG8, ANGPTL3, ANGPTL4, APOA1, APOA5, APOB, APOC2, APOE, CETP, GPIHBP1, LCAT, LDLR, LDLRAP1, LIPA, LIPC, LMF1, LPL, MTTP, and PCSK9. Second, genes associated with the patient’s broader phenotype, including ACE, APOA2, APOC3, CASR, CELSR2, CFTR, CPA1, CREB3L3, CTRC, CYP27A1, EPHX2, ESR1, F7, GCLC, GCLM, GHR, GPD1, HFE, ITGB3, MYLIP, NYNRIN, PRSS1, SAR1B, SCARB1, SLC22A1, SPINK1, SREBF2, and ST3GAL4. The second gene set includes loci associated with hypertriglyceridemia (HTG) in genome-wide association studies (GWASs) but does not comprise monogenic causal genes of lipid metabolism disorders. While certain genes, such as ANGPTL4 and APOC3, play established roles in modulating lipoprotein metabolism, they are not currently associated with specific monogenic syndromes.

The patient voluntarily provided biological samples and clinical data after providing written and verbal informed consent in the presence of a witness. Additional consent was obtained for the publication of de-identified case details. All patient information was anonymized and handled in strict confidentiality. The study protocol, including genetic analysis and data handling procedures, was reviewed, and approved by the Institutional Review Board (IRB) of the Faculty of Health Sciences of Indoamérica University in accordance with ethical standards for human research.

## 3. Results

**Case**. The patient was an Ecuadorian woman, mestizo, of 33 years old, born and living in Quito, Ecuador. No family history of lipid disorders nor fatty acid pathology was found in parents and children. There is no consanguinity or genetic trait that makes us suspect the pathology. In her personal history, it is reported that she showed acute pancreatitis secondary to severe hypertriglyceridemia in a recurrent way; she received lipid-lowering therapy with little adherence to treatment one year ago. There was occasional alcohol consumption without clinical complications.

The patient was treated at ICU of the Pablo Arturo Suárez Hospital in Quito, Ecuador, in 2022. The chief complaint was abdominal pain in the upper quadrant. The diagnostic evaluation at admission revealed increased leukocytosis, amylase, and lipase. Quantifying the creatinine levels, bilirubin, and coagulation times was impossible due to the presence of highly lipid serum. In the abdominal ultrasound, a distended gallbladder was observed, with a volume of 36.9 mL, thin walls, and acalculous. Although homogeneous, the pancreas showed increased echogenicity and peripancreatic laminar fluid. As this was the second episode of acute pancreatitis secondary to severe hypertriglyceridemia with no apparent cause, a disease of genetic origin was suspected.

The lipid profile on the first day showed high total triglycerides, high total cholesterol, low cholesterol HDL, and high cholesterol LDL. A previous lipid profile from when the patient was not sick is not available.

Normal valuesDay 1D2D3D7D11D22Total Triglycerides (mg/dL)Below 150 mg/dL2700 **↑**3700 **↑**423 **↑**333 **↑**245 **↑**107 **↑**Total Cholesterol (mg/dL)Below 200 mg/dL630 **↑**693 **↑**246 **↑**232 **↑**190 **↑**143 **↑**Cholesterol HDL (mg/dL)Above 60 mg/dL19 **↓**21 **↓**27 **↓**17 **↓**24 **↓**28 **↓**Cholesterol LDL (mg/dL)Below 100 mg/dL8087.495128 **↑**128 **↑**109.6 **↑**

The genetic diagnosis was made by genomic next-generation sequencing. It reported two heterozygous mutations of the LMF1 gene at positions chr16:879.570 (C>T variation) and chr16:870.819 (G>A variation). These are potentially deleterious variants of uncertain significance (VUS) and are also associated with hypertriglyceridemia. See Table 1 and Table 2. Clinically, the diagnosis was made using the Diagnostic Index of Suspicion for Familial CFS [12]. See Table 3.

**Figure 1:**
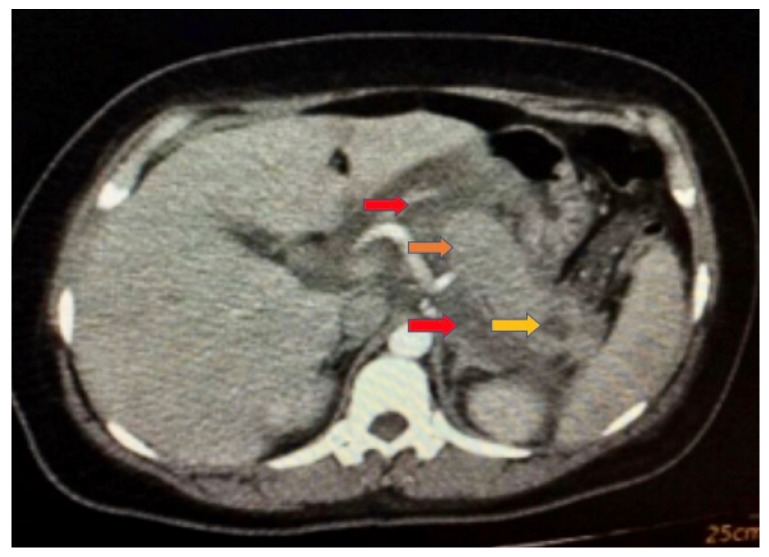
Simple Abdominal Tomography 72 h after admission: enlarged heterogeneous pancreas (orange arrow), with significant edema of peripancreatic fat (red arrows), and presence of a hypodense lesion in the pancreatic tail with irregular edges 16 × 9 mm with 5% necrotic area associated with peripancreatic collections (yellow arrow), cataloged as Balthazar D.

On day 2, she had hypercholesterolemia and hypertriglyceridemia, as well as normal high-density and low-density lipoproteins. On day 3, a decrease in hemoglobin was observed, although lipid values remained high. On day 7, she presented an exacerbation of pain, abdominal distension, and increased conjunctival jaundice. Low hemoglobin did remain, and hyperbilirubinemia was present, for which necro-hemorrhagic pancreatitis was suspected, for which she was transferred to the Intensive Care Unit. She was admitted to the ICU with SOFA 2, Marshall 2, BISAP 0, and APACHE 3, where she remained for 72 h and was sent to the floor in better conditions. In Simple Abdominal Tomography at 72 h, an enlarged heterogeneous pancreas was observed, with significant edema of peripancreatic fat and a hypodense lesion in the pancreatic tail. This lesion presented irregular borders 16 × 9 mm with a 5% necrotic area associated with peripancreatic collections, cataloged as Balthazar D. On day 11, there was a significant decrease in hemoglobin, accompanied by hyperbilirubinemia, an increased erythrocyte sedimentation rate, a reticulocyte production index of 1.5%, and elevated lactate dehydrogenase. The Coombs test was negative, while serum iron levels were decreased, transferrin remained within normal ranges, ferritin was elevated, and haptoglobin levels were normal. Inflammatory anemia was diagnosed, with positive markers for hemolysis such as haptoglobin at the lower limit of normal. On hospital day 22, the patient improved laboratory chemical levels and clinical symptoms. In subsequent outpatient controls, the patient maintained her clinical stability without new episodes of pancreatitis. No structural defects of the erythrocyte were found as a cause of anemia or subclinical hemolysis; pathologies of the erythrocyte membrane were ruled out through the osmotic fragility test without evidence of pathological hemolysis reproduced in the study, and hemoglobinopathies were also ruled out using electrophoresis studies. See Table 1 and Table 2 and Figure 1. Table 3 shows the distribution of clinical laboratory results through hospitalization. Table 4 shows the distribution of clinical laboratory results during hospitalization.

## 4. Discussion

Two LMF1 gene variants were identified: c.1142C>T (p.Pro381Leu) and c.897G>A (p.Gln299Gln). The Chr16:879,570 C>T variant (c.897G>A, ENST00000262301) has not been previously described in the literature and is found in 1 out of 122,000 individuals in the general population. In contrast, the Chr16:870,819 G>A variant (c.1142C>T, ENST00000262301) has been previously reported in the medical literature [9] and is detected in 1 out of 125,000 individuals worldwide. According to the American College of Medical Genetics and Genomics (ACMG) criteria [13], the c.1142C>T (p.Pro381Leu) variant is classified as a variant of uncertain significance (VUS). It meets moderate pathogenicity criteria due to its extremely low allele frequency in population databases (gnomAD); however, it is not listed in HGMD or ClinVar, and its clinical relevance remains undetermined. The c.897G>A (p.Gln299Gln) variant is also classified as a VUS due to limited supporting evidence. Although it has an extremely low frequency in gnomAD, computational analyses unanimously predict a deleterious effect on splicing, suggesting a potential pathogenic role. Notably, this variant has previously been reported in an Ecuadorian patient [14]. Additionally, a familial variant of Chr16:870,819 G>A (c.1142C>T, ENST00000262301) was identified in the patient’s son, who remains clinically asymptomatic. Although homozygous or heterozygous pathogenic variants in LMF1 can cause combined lipase deficiency (OMIM #246650), the clinical significance of the identified variants remains uncertain and warrants further investigation.

The 33-year-old female patient presented with a clinical phenotype consistent with familial chylomicronemia syndrome (FCS). However, due to the classification of the LMF1 variants as VUS, a definitive monogenic FCS diagnosis could not be confirmed. This case represents an intermediate phenotype, falling at the interface between monogenic and polygenic chylomicronemia. Functional studies are recommended to assess the potential impact of these variants on LPL activity and overall lipid metabolism.

This disorder is classified under the familial chylomicronemia syndrome spectrum (ICD-10 code E78.3; OMIM: 118830, 207750, 238600, 615947; ORPHA: 444490) and specifically falls under Familial LMF1 Deficiency (ORPHA: 535453). In the absence of molecular diagnostics, this case could have been attributed to a sporadic pattern of acquired episodic pancreatitis, rather than a genetically mediated disorder. LMF1 encodes a transmembrane protein localized in the endoplasmic reticulum, which plays a critical role in the post-translational maturation of lipoprotein lipase (LPL) and hepatic lipase (HL). Biallelic pathogenic variants in LMF1 result in severe LPL deficiency, moderately reduced HL activity, and persistent chylomicronemia. Two rounds of genetic analysis were performed. Initially, a familial chylomicronemia syndrome panel identified two mutations in the patient (mother). Subsequently, whole-exome sequencing (WES) was performed on whole blood samples from the patient and her son using next-generation sequencing (NGS). Data analysis incorporated REVEL (Rare Exome Variant Ensemble Learner), a predictive algorithm integrating multiple computational tools—including MutPred, FATHMM, VEST, PolyPhen, SIFT, PROVEAN, MutationAssessor, MutationTaster, LRT, GERP, SiPhy, phyloP, and phastCons—to assess missense variant pathogenicity. The results confirmed the presence of two familial VUS variants in both the patient and her clinically unaffected son. LPL deficiency should be suspected in individuals younger than 40 years of age presenting with recurrent acute pancreatitis, eruptive cutaneous xanthomas, and hepatosplenomegaly. Laboratory findings include severely impaired chylomicron clearance, resulting in lipemic plasma appearance and plasma triglyceride (TG) concentrations exceeding 2000 mg/dL, independent of fasting status. LPL enzymatic activity assays can provide diagnostic confirmation, although these tests are not routinely available and are primarily conducted in specialized laboratories.

Due to the heterogeneous nature of LPL pathogenic variants, genotype–phenotype correlations remain poorly established. Familial LPL deficiency is the most common monogenic form of familial chylomicronemia syndrome, previously known as Type I hyperlipoproteinemia. It has an estimated prevalence of 1 per million individuals in the United States, with Orphanet reporting a global prevalence of 1–9 per million. Based on this prevalence, there are an estimated 18 to 162 affected individuals in Ecuador, given a national population of 18 million. The disease has been described across diverse ethnic groups but exhibits increased prevalence in specific populations, such as Quebec, Canada, due to a founder effect. LPL deficiency typically manifests in childhood, with abdominal pain, recurrent pancreatitis, eruptive cutaneous xanthomas, and hepatosplenomegaly. Approximately 25% of affected children present symptoms before one year of age, with most cases occurring before age 10. However, some patients experience first-time presentation during pregnancy.

Disease severity correlates with chylomicron accumulation, and pancreatitis is the most severe clinical manifestation. The cornerstone of FCS management is a severely restricted low-fat diet (<20 g/day) to minimize chylomicron formation. The patient was managed with bowel rest, intravenous insulin therapy, lactated Ringer’s solution, and multimodal analgesia. Gemfibrozil (600 mg orally, twice daily) was prescribed as part of long-term lipid management. Acute chylomicronemia-associated pancreatitis is treated similarly to other forms of pancreatitis. Immediate cessation of dietary fat intake prevents further chylomicron production, while hypocaloric parenteral nutrition reduces hepatic VLDL synthesis. Excess caloric intake, particularly from carbohydrates, should be avoided to prevent hepatic triglyceride overproduction. Long-term lifestyle modifications include regular physical activity, caloric restriction, and alcohol and tobacco cessation. Medications that increase triglyceride levels—such as oral contraceptives, glucocorticoids, beta-blockers, and atypical antipsychotics—should be discontinued. For pregnant women with LPL deficiency, extreme dietary fat restriction (<2 g/day) is recommended during the second and third trimesters to maintain tight triglyceride control. Lipid-lowering pharmacotherapies, including fibrates, niacin, and statins, are ineffective in monogenic LPL deficiency.

## 5. Conclusions

This is a new Ecuadorian report of LMF1-related hypertriglyceridemia, highlighting the need for functional studies to confirm pathogenicity. Given the classification of both variants as VUS, further research is required to elucidate their clinical significance. This case underscores the necessity of a combined genetic and biochemical approach for diagnosing and managing severe hypertriglyceridemia.

## Figures and Tables

**Table 1:** Patient’s mutations found; 1st round. Familial chylomicronemia syndrome panel (FMS) *.

Transcript Gene	Position	Variation	Consequence	Zygosity	Classification
*LMF1* (NM_022773.4)	chr16:879.570	C > T	p.Gln299=ENST00000262301	Heterozygous (1 copy)	Variant of uncertain significance (VUS)
*LMF1* (NM_022773.4)	chr16:870.819	G > A	p.Pro381Leu=ENST00000262301	Heterozygous (1 copy)	Variant of uncertain significance (VUS)

* Performed by Mendelics Analysis Genomics laboratory (https://mendelics.com.br/en/).

**Table 2:** Patient’s mutations found; 2nd round. Clinical exome, sequencing, whole blood, by next-generation sequencing (NGS) *.

Transcript Gene	DNA Protein, Reference SNP ID	MAF gnomAD	ClinVar HGMD Literature	Zygosity	Pathology Related	Inheritance Pattern	Meta Predictor REVEL **
Patient’s mutations							
LMF1 (NM_022773.4)	c.897G>A p.(Gln299Gln) -	Not available	None CM224509	Heterozygous	Combined deficiency of lipase (MIM_246650)	Autosomal recessive	Variant of uncertain significance (VUS)
LMF1 (NM_022773.4)	c.1142C>T p.(Pro381Leu) rs1408203743	0.000004038	None	Heterozygous	Combined deficiency of lipase (MIM_246650)	Autosomal recessive	Benign
Patient son mutation							
LMF1 (NM_022773.4)	c.1142C>T p.(Pro381Leu) rs1408203743	0.000004038	None	Heterozygous	Combined deficiency of lipase (MIM_246650)	Autosomal recessive	Benign Familial variant

* Performed by SYNLAB International GmbH (https://www.synlab.com/); ** REVEL (rare exome variant ensemble learner), an ensemble method for predicting the pathogenicity of missense variants based on individual tools.

**Table 3:** Diagnostic index of suspicion for familial chylomicronemia syndrome [12].

Variable	Points
Fasting serum triglycerides greater than 885 mg/dL (three consecutive measurements)	+5
Fasting serum triglycerides greater than 1760 mg/dL (at least once)	+1
Previous measurement of triglycerides less than 175 mg/dL	−5
Absence of secondary causes (no pregnancy and estrogen use)	+2
History of pancreatitis	+1
Recurrent unexplained abdominal pain	+1
No response to lipid-lowering treatment	+1
No history of familial combined hyperlipidemia	+1
The onset of symptoms according to age:	
Less than 40 years	+1
Less than 20 years	+2
Less than ten years	+3

Interpretation: For values of 10 or more, the diagnosis is very likely, 8 to 9 unlikely, and less than 8 unlikely. The patient scored 10 points.

**Table 4:** Distribution of clinical laboratory results during hospitalization.

	D1	D2	D3	D7	D11	D22
Hemoglobin (g/dL)	17.4	16.6	13.1	9.9	10.6	13.5
Hematocrit (%)	39.9	49.6	38.3	30.6	32.8	41.9
MCV (fL)	84.3	84.2	88.0	88.3	90.1	86.0
WBC (×10^9/^L)	13.8	12.9	13.7	9.4	5.4	5.1
Neutrophils (×10^9/^L)	11.8	11.3	12.0	7.7	3.3	2.1
Platelet (×10^9/^L)	223	237	180	206	309	212
Reticulocytes (%)	-	-	-	-	2.9	-
Ferritin (ng/mL)	-	-	-	-	956	-
LDH (U/L)	-	-	-	-	662	-
Haptoglobin (Ug/dL)	-	-	-	-	36	-
Creatinine (mg/dL)	lipemic serum	0.5	0.4	0.4	0.5	0.7
Coombs direct	-	-	-	-	Neg.	-
C reactive protein (mg/dL)	-	-	31.8	26	-	-
Bilirubin total (mg/dL)	lipemic serum	0.6	0.8	2.2	0.8	-
Bilirubin direct (mg/dL)	lipemic serum	0.2	0.2	0.7	0.5	-
Bilirubin Indirect(mg/dL)	lipemic serum	0.4	0.6	1.5	0.3	-
Amylase (mg/dL)	480	221	90	44	-	-
Lipase (mg/dL)	984	446	233	66	-	-
Cholesterol total (mg/dL)	-	693	246	232	-	143
Cholesterol HDL (mg/dL)	-	21	27	17	-	28
Cholesterol LDL (mg/dL)	-	87.4		128	-	109.6
Triglycerides (mg/dL)	-	3700	423	333	-	107

## Data Availability

The data supporting this manuscript are available upon request to the corresponding author and patient approval.
